# Comparative Genomics in Perennial Ryegrass (*Lolium perenne* L.): Identification and Characterisation of an Orthologue for the Rice Plant Architecture-Controlling Gene *Os*ABCG5

**DOI:** 10.1155/2011/291563

**Published:** 2011-09-15

**Authors:** Hiroshi Shinozuka, Noel O. I. Cogan, German C. Spangenberg, John W. Forster

**Affiliations:** ^1^Biosciences Research Division, Department of Primary Industries, Victorian AgriBiosciences Centre, La Trobe University Research and Development Park, 1 Park Drive, Bundoora, VIC 3083, Australia; ^2^Molecular Plant Breeding Cooperative Research Centre, Bundoora, VIC 3083, Australia; ^3^Dairy Futures Cooperative Research Centre, Bundoora, VIC 3083, Australia; ^4^La Trobe University, Bundoora, VIC 3086, Australia

## Abstract

Perennial ryegrass is an important pasture grass in temperate regions. As a forage biomass-generating species, plant architecture-related characters provide key objectives for breeding improvement. *In silico* comparative genomics analysis predicted colocation between a previously identified QTL for plant type (erect versus prostrate growth) and the ortholocus of the rice *Os*ABCG5 gene (*Lp*ABCG5), as well as related QTLs in other Poaceae species. Sequencing of an *Lp*ABCG5-containing BAC clone identified presence of a paralogue (*Lp*ABCG6) in the vicinity of the *Lp*ABCG5 locus, in addition to three other gene-like sequences. Comparative genomics involving five other 5 grass species (rice, *Brachypodium*, sorghum, maize, and foxtail millet) revealed conserved microsynteny in the ABCG5 ortholocus-flanking region. Gene expression profiling and phylogenetic analysis suggested that the two paralogues are functionally distinct. Fourteen additional ABCG5 gene family members, which may interact with the *Lp*ABCG5 gene, were identified through sequencing of transcriptomes from perennial ryegrass leaf, anther, and pistils. A larger-scale phylogenetic analysis of the ABCG gene family suggested conservation between major branches of the Poaceae family. This study identified the *Lp*ABCG5 gene as a candidate for the plant type determinant, suggesting that manipulation of gene expression may provide valuable phenotypes for perennial ryegrass breeding.

## 1. Introduction

Perennial ryegrass is an economically important temperate pasture grass species and a diploid (2*n* = 2*x* = 14) member of the Poaceae family which includes other major cereal crops such as rice (*Oryza sativa* L.), wheat (*Triticum aestivum* L.), barley (*Hordeum vulgare* L.), sorghum (*Sorghum bicolor* L.), and maize (*Zea mays* L.) [1, 2]. Due to superior herbage digestibility and grazing tolerance, perennial ryegrass has been a primary target for molecular breeding among forage and turf crops [3]. Following establishment of a whole-genome genetic map consisting of 7 linkage groups (LGs) [4, 5], a number of quantitative trait loci (QTLs) related to variation for herbage yield and quality have been identified [6–9]. Plant morphological traits contribute to such variation in pasture grass species and are largely controlled by genetic factors [6, 10]. For example, evaluation of a one-way pseudotestcross genetic mapping population obtained a broad sense heritability (*H*) value of 0.73 for the herbage fresh weight character, and significant correlations were observed between this and other traits such as plant height and tiller number [6]. Improvement of forage productivity and quality may hence be accomplished through breeding-based manipulation of plant architecture-related traits.

Comparative genomics provides a strategy for utilisation of sequence colinearity between two or more related species for molecular genetic studies [11]. The completion of whole-genome sequencing studies for the model grass species *Brachypodium distachyon* (L.) Beauv, in addition to those of the cereals rice, sorghum, and maize, revealed high-levels of gene order conservation between Poaceae species despite lineage divergences dating over 40 million years ago (MYA) [11–13]. Such gene-based microsynteny can expedite comparative genetics approaches for gene identification and trait dissection. For example, a candidate gene for the semidwarfing (*sdw1/denso*) mutation of barley was identified through comparative mapping with rice [14]. Physical mapping of another barley semi-dwarf locus (*sdw3*) has also been progressed using microsynteny between the genome sequences of rice, *Brachypodium sylvaticum* (Huds.), P. Beauv., and sorghum [15]. Recently, perennial ryegrass has been subjected to comparative genetic analysis. Assignment of gene-associated genetic markers permitted comparison between the genetic linkage maps of perennial ryegrass, rice, wheat, and oat (*Avena sativa* L.), macrosynteny between orthologous LGs of each species [4]. Candidate genes for heading date, herbage quality, and disease resistance QTLs were also identified through comparative approaches [7, 8, 16, 17]. A partial physical map was generated in order to support map-based cloning strategies for the identification of the perennial ryegrass gametophytic self-incompatibility (SI) loci through establishment of microcolinearity with the rice genome in the target regions [18]. 

Restriction of tiller number per plant is a key objective to maximise grain yield in rice breeding. A member of the ATP-binding cassette protein subfamily G (ABCG), gene family *Os*ABCG5, was identified as a tiller number determinant [19]. Although a total of 50 rice gene loci are predicted to encode ABCG proteins, none had been functionally characterised until relatively recently [19, 20]. A mutant genotype with a single nonsynonymous substitution in the *Os*ABCG5 gene resulted in phenotypes with significantly reduced height and tiller number as compared to nonmutant comparators [21]. Further characterisation suggested that the gene controls formation of tiller buds independently of the plant growth regulator auxin, and that transcripts were localised in vascular cells and epidermis of older leaves, as well as the root endodermis, pericycle, and stele [19]. Further study of *Os*ABCG5 has potential for application in the breeding of elite high-yielding rice varieties. Conversely in pasture grasses, for which profuse tillering is a desirable character, identification and manipulation of the orthologous gene could be highly valuable.

The ABCG gene family encodes proteins which have been extensively studied in humans as multidrug resistance transporters and disorder-related molecules [22]. ABCG proteins, which are composed of nucleotide-binding (NBD) and transmembrane domains (TMD), are divided into half- and full-size groups [20]. Half-size ABCG transporters are known to function as homodimers and/or heterodimers [22]. In the model plant *Arabidopsis thaliana* (L.) Heynh., the *At*ABCG12 protein, which is a half-size transporter, forms a heterodimer with the *At*ABCG11 protein to export cuticular lipids [23]. Since the *Os*ABCG5 gene belongs to the half-size gene cluster, the translated product may also require a partner ABCG transporter to form a heterodimer [19].

Transcriptome sequencing based on massively parallel DNA sequencing technologies is an emerging method for systematic discovery of genes associated with specific developmental stages or environmental conditions [24, 25]. A large quantity of sequence information has been collected from nonmodel species, and the resulting data sets may be used for further genetic studies [26]. The method is particularly suitable for survey of complex gene families in which differential expression may occur, such as those encoding ABCG proteins. As an example, perennial ryegrass, candidate genes for selenium transport have been identified through use of transcriptome sequencing [27].

In this study, *Lp*ABCG5, the putative orthologue of the *Os*ABCG5 gene in perennial ryegrass, has been identified and characterised. The *Lp*ABCG5 gene colocates with a previously identified QTL for plant architecture, and allelic diversity at the locus may be responsible for quantitative variation for this trait. Sequencing of a bacterial artificial chromosome (BAC) clone containing the *Lp*ABCG5 sequence revealed the presence of a closely adjacent paralogous gene, designated *Lp*ABCG6. Additional ABCG gene family candidates have been identified through transcriptome sequencing. Computational analysis indicates that the molecular function of *Os*ABCG5 gene orthologues is broadly conserved within the Poaceae family. Modification of *Lp*ABCG gene expression may provide novel phenotypes for the breeding of both pasture-type and turf-type ryegrasses.

## 2. Materials and Methods

### 2.1. *In Silico* Genomic Analysis

DNA sequence information was obtained from the NCBI (http://www.ncbi.nlm.nih.gov/), *Brachypodium distachyon* (http://www.brachypodium.org/), GrainGene (http://www.gramene.org/), and Phytozome (http://www.phytozome.net/) databases. Gene structure prediction was performed using the FGENESH program with the parameter setting for monocot plants (http://linux1.softberry.com/berry.phtml). Phylogenetic analysis was performed using the CLUSTALW program (http://www.genome.jp/tools/clustalw/). Nonsynonymous and synonymous nucleotide substitution rates (*K*_*a*_ and *K*_*s*_, resp.) were calculated using the Synonymous Non-synonymous Analysis Program (SNAP; http://www.hiv.lanl.gov/) [28, 29]. Regression lines were deduced using the substitution rates from the ABCG5 genes and the following estimated elapsed times since evolutionary divergence: 53 MYA for species in the Panicoideae (sorghum and maize) and those in Ehrhartoideae and Pooideae subfamilies of the Poaceae; 47 MYA for Ehrhartoideae (rice) and Pooideae (*Brachypodium*, barley and perennial ryegrass), 35 MYA between *Brachypodium* and the other Pooideae species; 21 MYA between barley and perennial ryegrass, and 12 MYA between sorghum and maize. The elapsed times were calculated as averages between the minimum and maximum values of estimated periods since divergence [12]. The time of divergence between barley and perennial ryegrass was estimated from a previous study involving a closely related Poeae grass species, tall fescue (*Festuca arundinacea* Schreb. syn, *Lolium arundinaceum*) [30].

### 2.2. Genetic Linkage Analysis

PCR primers (ABCG5_f: 5′-TCGTGCTCAAGTTCACCGAC-3′, ABCG5_r: 5′-GGAGAACATGAGCGTCTCCT-3′) were designed based on consensus sequence information from the *Os*ABCG5 gene and putative orthologues from barley, wheat, and *Brachypodium*. The p150/112 sib-ship, which is a one-way pseudotestcross population, was used for genetic mapping [5, 31]. PCR amplicons from the population were sequenced using the ABI 3730xl prism automatic sequencer (Applied Biosystems at present Applera, Foster City Calif, USA) to identify single nucleotide polymorphisms (SNPs) between haplotypes from the multiply-heterozygous parental genotype (C3) [4, 5]. *Lp*ABCG5 locus-specific primers (*Lp*ABCG5_f: 5′-ATCAGGAAGGAGAGCCTCCA-3′, *Lp*ABCG5_r: 5′-ATGATGGTGTTGGCCGCGTT-3′) and a single nucleotide primer extension (SNuPe) primer (SNP_*Lp*ABCG5: 5′-GCAAGGAGAAGAAGAA-3′) were designed to detect adenosine- (A-) guanine (G) substitution SNP, and members of the p150/112 sib-ship were genotyped by the SNuPe assay with use of SNuPe premix (GE Healthcare, Chalfont St. Giles, Buckinghamshire, UK) and the MegaBACE 4000 instrument (GE Healthcare). The JoinMAP 3.0 application was used for genetic map construction [32].

### 2.3. BAC Clone Sequencing

The perennial ryegrass BAC-based genomic library [3, 33] was PCR-screened using *Lp*ABCG5 gene-specific primers. A Sanger shotgun sequencing library was prepared using the Large-Construction Kit (QIAGEN, Hilden, Germany) and TOPO Shotgun Subcloning Kit (Invitrogen, Carlsbad, Calif, USA). The resulting *E. coli* colonies with 1-2 kb DNA inserts were used for multiple displacement amplification (MDA) reactions with the TempliPhi kit (GE Healthcare) [34]. The inserted DNA was sequenced using the ABI 3730xl sequencer, and sequence reads were assembled using Sequencher software (Gene Codes, Mich, USA).

### 2.4. mRNA Extraction and cDNA Synthesis

Root, crown, leaf, inflorescence, anther, and pistil tissues were harvested from specific perennial ryegrass genotypes (Aurora_6_ (AU_6_) and Impact_04_) which were established at DPI-Hamilton (Victoria, Australia) [35]. The RNeasy Plant Mini Kit (QIAGEN) was used for the total RNA purification process, and contaminating genomic DNA was eliminated by the on-column DNase digestion method using DNase I (QIAGEN). A total of 150 ng extracted RNA samples were used for cDNA synthesis with the SMART PCR cDNA synthesis Kit (Clontech, Terra Bella Avenue, Calif, USA).

### 2.5. Digital Gene Expression Profiling

cDNA samples from root, crown, leaf, and inflorescence tissues of the AU_6_ genotype were used for gene expression profiling. PCR amplification was performed using 5 ng aliquots of cDNAs as DNA templates in concert with gene-specific PCR primer pairs for the *Lp*ABCG5, *Lp*ABCG6 (*Lp*ABCG6_f: 5′-TAACGCTCAACGGGGACG-3′, *Lp*ABCG6_r: 5′-TCGTCGCCGATGATGGTGTT-3′), and perennial ryegrass glyceraldehydes 3-phosphate dehydrogenase (GAPDH) gene (*Lp*GAPDH_f: 5′-TGGTGCCAAGAAGGTCATCAT-3′, *Lp*GAPDH_r: 5′-GACCATCAACAGTCTTGG-3′) [36]. PCR amplicons were visualised on 2.5% (w/v) agarose gels stained with SYBR Safe (Invitrogen).

### 2.6. Transcriptome Analysis Using the GS FLX Platform

RNA samples derived from leaves of the Imapct_04_ genotype and anthers and pistils of the AU_6_ genotype were prepared for cDNA library construction with the modified 3′-cDNA synthesis primer (5′-AAGCAGTGGTATCAACGCAGAGTCGCAGTCGGTACTTTTTTCTTTTTV-3′) and the Advantage 2 PCR Kit (Clontech) [37]. The cDNA libraries were normalised using the TRIMMER cDNA Normalization Kit (Evrogen, Miklukho-Maklaya st, Moscow, Russia) and sequenced using the GS FLX pyrosequencing instrument with the Titanium chemistry (Roche Diagnostic, Basel, Switzerland) following the product manual. The successful sequence reads were assembled through use of the Newbler program (Roche Diagnostics).

## 3. Results and Discussion

### 3.1. *In Silico* Comparative Genetics and Empirical Mapping of the *Lp*ABCG5 Gene

**Figure 1:**
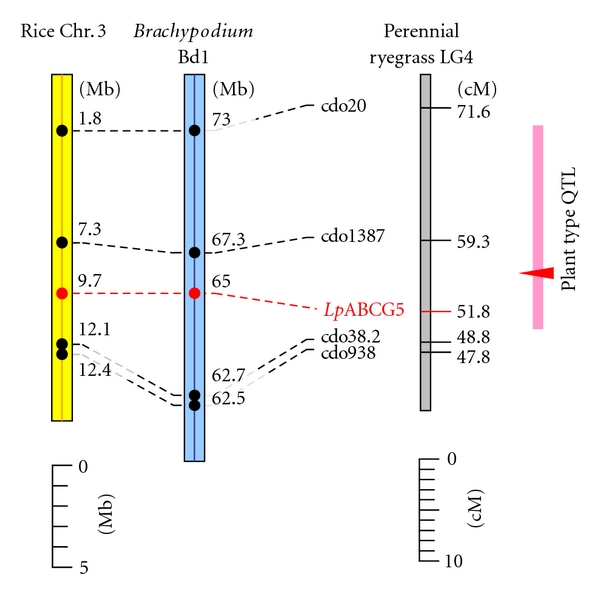
*In silico* comparative and empirical genetic mapping of the Poaceae ABCG5 locus. The gray bar represents the plant type QTL-containing region of perennial ryegrass LG4, and the yellow and blue bars show putative orthologous regions in rice and *Brachypodium*, respectively. The positions of the QTL interval and maximum LOD value are indicated with the pink line and red triangle, respectively. The putative ortholoci are connected with the broken lines, and the locations of loci are shown on the right side of maps.

Through *in silico* mapping (Figure 1), macrosyntenic relationships were identified between a segment of rice chromosome 3 and a region of perennial ryegrass LG4 previously shown to contain a QTL for plant type. These results were consistent with previous studies [4]. A total of 4 functionally associated loci (xcdo20, xcdo1387, xcdo38, and xcdo938) were identified close to the QTL, based on use of oat cDNAs used as hybridisation probes to detect restriction fragment length polymorphisms (RFLPs) [4]. DNA sequences similar to the oat cDNAs were identified in the 1.8–12.4 Mb region of rice chromosome 3. Conservation of the same gene order was also revealed in chromosome Bd5 of *B. distachyon* (62.5–73 Mb) (Figure 1). The rice and *Brachypodium *ABCG5 orthologues were confirmed as locating in the interval between the cdo1387 and cdo38 ortholoci of each species. 

PCR amplification based on the Poaceae ABCG5 consensus primers generated DNA fragments c. 250 bp in length from the C3 parental genotype, and subsequent analysis revealed sequences derived from two types of amplicon. The majority class showed the highest similarity among rice unigenes to the *Os*ABCG5 sequence, while the minor class bore the same relationship to the paralogous *Os*ABCG6 gene (Table 1) [20]. The respective sequences were designated *Lp*ABCG5 and *Lp*ABCG6. An SNP marker for the *Lp*ABCG5 sequence was designed, and a total of 47 genotypes of the p150/112 population were successfully genotyped, resulting in a segregation ratio of 23 : 24, close to 1 : 1 expectation. The *Lp*ABCG5 locus was mapped to the interval between the cdo1387 and cdo38 markers on LG4, providing increased confidence for the predicted comparative relationships with model species in this region of the genome.

The plant type phenotypic character was previously evaluated in the p150/112 population based on semiquantitative visual score scale from 1 (most erect) to 9 (most prostrate) [6]. The broad sense heritability for this trait was 0.83, and the maximum log-of-odds (LOD) value for the QTL probability distribution was observed in the cdo1387-cdo38 interval marker loci (Figure 1) [6]. Plant type variation in the p150/112 population was significantly correlated with both plant height (maximum length in cm from the base to the top of the plant) and tiller number, revealing a tendency for prostrate individuals to exhibit short plant height and low tiller numbers [6]. Rice plants with the putative nonfunctional *Os*ABCG5 allele also displayed reduction of both plant height and tiller number phenotypic characters [19, 21]. Colocation between the *Lp*ABCG5 candidate gene and a plant type QTL suggests the possibility of functional conservation between rice and perennial ryegrass ABCG5 orthologues [19, 21]. Although tiller number and plant height phenotypic characters were also examined in the previous study, no significant locus for tiller number was identified, and plant height QTLs were only detected on LGs 1 and 3 [6]. As the QTL analysis was based on comparison of effects due to segregation of two parental alleles within a biparental population [38], it is possible that effects of the specific *Lp*ABCG5 alleles on tiller number and plant height determinates were too small to be detected as significant. Association mapping experiments, which potentially involve a larger number of alleles for each locus, may be capable of detecting larger effects of *Lp*ABCG5 allelic diversity on the relevant characters.

Further support for the hypothesis of gene functional conservation comes from studies of the closely related taxon Italian ryegrass (*Lolium multiflorum* Lam.), for which a plant height QTL was detected in close linkage to the cdo38 and cdo1387 marker loci [10]. In barley, plant height and ear number QTLs have also been located on the long arm of chromosome 4H, which has a macrosyntenic relationship with the lower part of perennial ryegrass LG4 [4, 39, 40]. Further comparative analyses are capable of refining the potential causal relationships between *Os*ABCG5 gene orthologues and plant architecture-related QTLs in these grass species.

### 3.2. Screening and Sequencing of the *Lp*ABCG5 BAC Clone

Two candidate BAC clones were identified from the perennial ryegrass genomic library, and one, designated *Lp*BAC39-B03, was subjected to further analysis. A total of 653 Sanger reads (>300 bp) were generated by the shotgun sequencing method, and the assembly process created 19 large (>2.5 kb) sequence contigs. The *Lp*ABCG5 and *Lp*ABCG6 genomic sequences were identified in two contigs of 10.4 kb and 7.6 kb length (NCBI acc. no. JN051254 and JN051255), respectively. In other contigs, LOC_Os03g17300-, LOC_Os03g17310, and LOC_Os03g17340-like sequences (NCBI acc. no. JN051256, JN051257 and JN051258), all predicted to be conserved within this genomic region, were identified by the BLASTN search (Table 1). 

**Figure 2:**
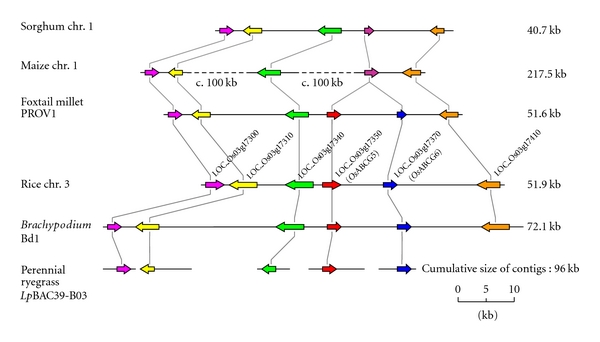
Comparative physical map for the vicinity of the Poaceae ABCG5 locus. The horizontal black lines represent genomic sequences of Poaceae species. The arrows represent gene(like) sequences, indicating direction of transcription, and putative orthologues are shown in complementary colours; ABCG5 orthologues in red; ABCG6 orthologues in blue; and ABCG gene-like sequences in sorghum and maize in purple. Physical lengths (in kb) are shown on the right side of maps.

A comparative physical map for the vicinity of the ABCG5 locus was generated, based on genomic sequence information from perennial ryegrass, rice, *Brachypodium*, sorghum, maize, and foxtail millet (*Setaria italica* (L.) P. Beauv.) (Table 1, Figure 2). Only one ABCG gene-like sequence was identified in the corresponding regions of sorghum and maize, while two ABCG gene-like sequences were found in the other species. The relative order of the two paralogues was conserved for those species in which contiguous sequence assembly was completed. These results support classification of the *Lp*ABCG5 and *Lp*ABCG6 genes as orthologues of corresponding rice genes. Protein coding regions composed of single exons were predicted for the *Lp*ABCG5 (2382 bp) and *Lp*ABCG6 (1977 bp) genes, respectively.

### 3.3. Phylogenetic Analysis for ABCG5 and ABCG6 Genes

**Figure 3:**
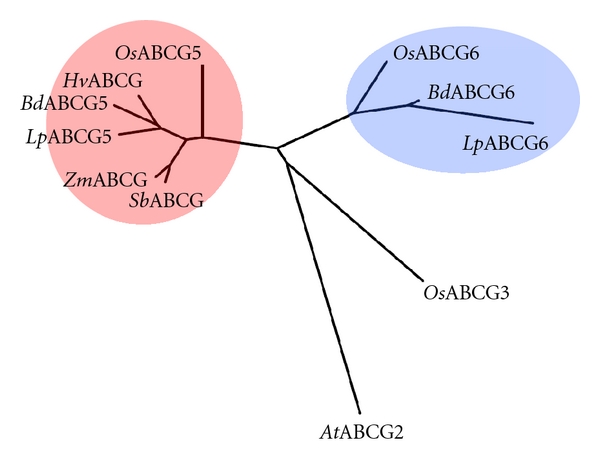
Phylogenetic trees of ABCG5 and ABCG6 orthologues. Clusters including the *Os*ABCG5 and *Os*ABCG6 genes are shown in red and blue, respectively. The barley, *Brachypodium*, sorghum, and maize ABCG genes are indicated with *Hv*, *Bd*, *Sb,* and *Zm* prefixes, respectively.

A phylogram was constructed to include the ABCG5 and ABCG6(like) genes of monocot species and of *Arabidopsis* (Figure 3). The *Lp*ABCG5 sequence showed the highest sequence similarity to the *At*ABCG2 and *Os*ABCG3 genes, following exclusion of the *Os*ABCG5 and *Os*ABCG6 genes. For barley, maize, and sorghum, only single cDNA sequence database accessions were identified. In the tree, the genes from barley, sorghum and maize were clustered together with the ABCG5-like sequences from rice, *Brachypodium,* and perennial ryegrass, while the rice, *Brachypodium,* and perennial ryegrass ABCG6-like sequences formed a separate cluster. The *Os*ABCG3 and *At*ABCG2 genes, which were classified in the same group as *Os*ABCG5 and *Os*ABCG6 in the previous study, were not clustered with either ABCG5 or ABCG6 genes [20]. This result suggests that the ABCG-like genes observed in sorghum and maize are orthologues of *Os*ABCG5, but not *Os*ABCG6. In the previous report, few putative orthologous pairs were identified when ABCG gene family members in *Arabidopsis* and rice were subjected to phylogenetic analysis, suggesting a taxon-specific functional diversification scenario for the gene family [20]. In contrast, the results of the present study suggest functional conservation of each ABCG5 and ABCG6 orthologous group within the Poaceae family. A relatively large genetic distance between the ABCG5 and ABCG6 clusters may reflect distinct molecular functions for the two groups.

### 3.4. Evolutionary Genetic Analysis

**Figure 4:**
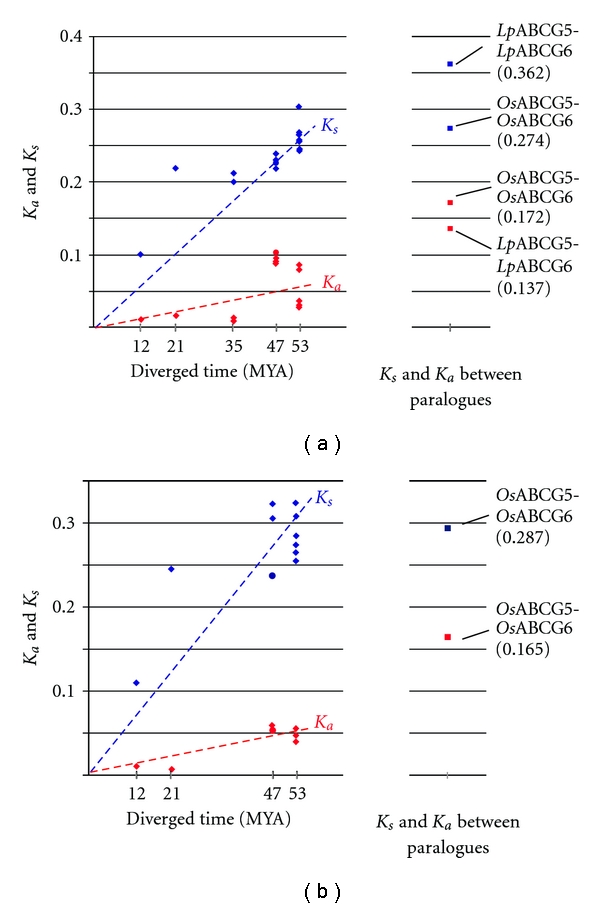
*K*_*a*_ and *K*_*s*_ values for the NBD (a) and TMD (b) peptide-coding regions. The *K*_*a*_ (red) and *K*_*s*_ (blue) values are plotted according to the estimated elapsed times since lineage divergence. The diamond (ABCG5) and circle (ABCG6) symbols denote substitution ratios between orthologous combinations, while square symbols denote substitution ratios between paralogous combinations. Broken lines represent regression plots.

Due to the presence of insertion/deletion (indel) polymorphism, *K*_*a*_ and *K*_*s*_ values could not be adequately determined when whole coding region sequences were used as query data. DNA sequences for the NBD (795 bp, including a 6 bp indel) and TMD (597 bp) peptide-coding regions of the ABCG proteins were used as individual queries, due to relatively high sequence conservation. The *K*_*a*_ and *K*_*s*_ values between orthologous sequences were plotted according to estimated elapsed time (MYA) since divergence of species (Figure 4) [12, 30]. The means and standard deviations for the most distantly related ABCG5 orthologue pairs, which diverged when the BEP (Bambusoideae, Ehrhartoideae, and Pooideae) and PACC (Panicoideae, Arundinoideae, Centothecoideae, and Chloridoideae) clades (representing groups of subfamilies) branched (45–60 MYA), were 0.047 ± 0.023 (*K*_*a*_, NBD), 0.046 ± 0.006 (*K*_*a*_, TMD), 0.244 ± 0.018 (*K*_*s*_, NBD), and 0.284 ± 0.026 (*K*_*s*_, TMD). The values for the comparison of paralogous genes (e.g., *O*sABCG5 and *Os*ABCG6) were also examined (Figure 4). Both *K*_*a*_ and *K*_*s*_ values for paralogues were higher than the average values obtained for orthologue comparisons. The substitution rates between the* At*ABCG2 and monocot ABCG5 genes were also analysed. *K*_*a*_ values of 0.185 ± 0.005 (NBD) and 0.181 ± 0.007 (TMD), and *K*_s_ values of 0.902 ± 0.021 (NBD) and 0.845 ± 0.005 (TMD) were obtained, all higher than those obtained from comparison of ABCG5 and ABCG6 paralogues. These comparisons of nucleotide substitution frequencies suggest that the two Poaceae ABCG paralogues resulted from duplication of the ancestral gene, which occurred prior to divergence of the BEP and PACC clades. Although the common ancestor of contemporary grass species possessed two copies, the ABCG6 gene was probably deleted from the genome of the common ancestor of sorghum and maize after lineage divergence within the Panicoideae subfamily, as two genes are present in foxtail millet (supertribe Paniceae), but only the ABCG5 gene was detected in sorghum and maize (supertribe Andropogoneae) (Figure 2).

### 3.5. Gene Expression Analyses

**Figure 5:**
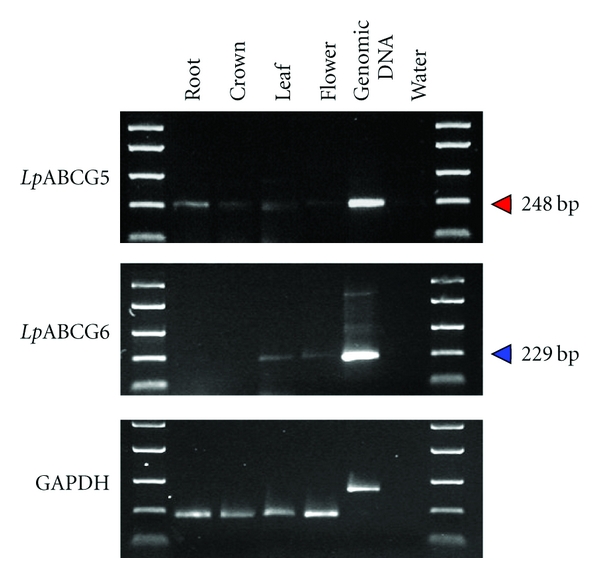
Gene expression profiling for the *Lp*ABCG5 and *Lp*ABCG6 genes. The fragment sizes are shown with triangles. The EasyLadder I (BIOLINE) was used as a size standard. Genomic DNA (AU_6_) and distilled water were used as experimental controls. The PCR primers for the GAPDH gene were designed across intron sequence, in order to identify spurious products due to genomic DNA contamination in the cDNA samples.

PCR assays using cDNA templates indicated that the *Lp*ABCG5 gene is expressed in all examined tissues, while the expression of *Lp*ABCG6 gene is restricted to leaves and inflorescences (Figure 5). Expression of the *Os*ABCG5 gene was confirmed in rice roots and basal shoots by Northern hybridization analysis, but no signal was detected from either leaf blades or panicles [19]. As different detection technologies were used in each study, a conclusion of differential gene expression between species is not definitive. Further detailed analysis using common methodology would be required for comparison of expression patterns between the orthologous genes. However, the differences in expression patterns of the *Lp*ABCG5 and *Lp*ABCG6 genes do support the hypothesis, derived from the phylogenetic study, that the two genes are functionally distinct.

### 3.6. Transcriptome Sequencing Analysis

Totals of c. 57,000, 63,000 and 116,000 reads were obtained from pyrosequencing analysis of perennial ryegrass anther-, pistil-, and leaf-derived cDNA libraries, of which the average lengths were c. 233, 291 and 300 bp, respectively. After assembly, 1,315, 2,151, and 5,867 contigs were generated and c. 46,000, 48,000, and 26,000 reads remained as singletons in each library. A total of 14 fragments were identified as sharing sequence similarity with members of the rice ABCG gene family (Table 2). As both *Lp*ABCG5 and *Lp*ABCG6 genes are expressed in leaf and inflorescence tissues, some of the newly identified ABCG-like genes might be interacting partners of the two genes, due to coexpression pattern. However, orthologues for the *Os*ABCG3, *Os*ABCG10, *Os*ABCG11, and *Os*ABCG21 genes, which have been described as candidate heterodimer partners of the *Os*ABCG5 gene, were not identified [19]. Sequence reads representing the *Lp*ABCG5 and *Lp*ABCG6 genes were not found within the datasets, suggesting that the transcriptome analysis was still at insufficient depth to identify all mRNA types. A larger-scale sequencing analysis is hence required to obtain a full set of expressed ABCG genes from perennial ryegrass leaf and inflorescence tissues.

### 3.7. Phylogenetic Analysis for the ABCG Gene Family in Poaceae

**Figure 6:**
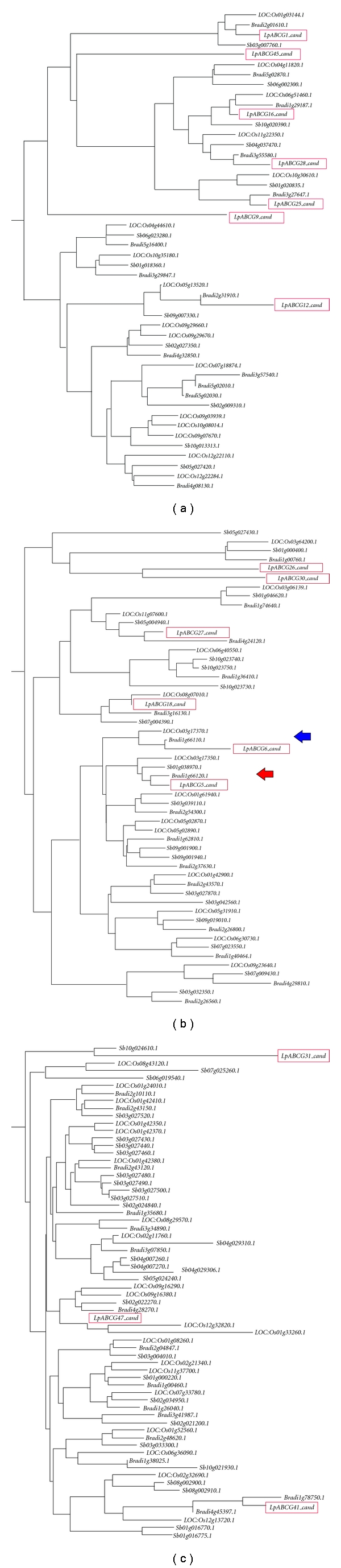
Phylogenetic trees of the ABCG gene family of the Poaceae species. The rice half- ((a) and (b)) and full-size (c) ABCG transporter genes are separately clustered. The rice, *Brachypodium*, sorghum, and perennial ryegrass genes are indicated with *LOC:Os*, *Bradi*, *Sb,* and *Lp* prefixes, respectively. ABCG transporter sequences from perennial ryegrass are highlighted with red boxes. The clusters including the ABCG5 and ABCG6 genes are indicated with the red and blue arrows, respectively (b).

Totals of 43 and 58 ABCG gene-like coding sequences (CDSs) were identified from *Brachypodium* and sorghum, respectively. A total of 167 sequences were subjected to phylogenetic analysis. Preliminary analysis indicated that the ABCG orthologues can be divided into 3 sub-groups: two of half-size and one of full-size transporters (Figure 6). Of 14 perennial ryegrass sequences obtained from transcriptome sequencing, 7 were classified into the first group, while 4 and 3 were categorised in the second and third groups, respectively. The *Lp*ABCG5 and *Lp*ABCG6 sequences clustered with their orthologues in the second group. In the phylograms, 7 ABCG-like fragments derived from transcriptome sequencing clustered with putative rice orthologues identified by BLASTN similarity search, while the other 7 were more distantly related to the predicted orthologues. The *Lp*ABCG18 and *Lp*ABCG 27 candidate sequences were relatively closely related to the *Lp*ABCG5 and *Lp*ABCG6 genes, respectively. However, the analysis suggested the presence of more closely related paralogues in perennial ryegrass, such as the orthologue of LOC_Os01g61940 (*Os*ABCG3).

The majority of half-size transporter genes formed small clades with 2-3 sequences from other species, suggesting mutual orthologous relationships (Figures 6(a) and 6(b)). A minority of genes, in contrast, formed such clades in the full-size transporter group (Figure 6(c)), suggesting different patterns of evolutionary divergence between the two types of ABCG genes. A total of 31 clades composed of putative orthologues were identified in the phylograms. Some paralogues that are closely adjacent in physical terms were also near-neighbours in sequence terms (e.g., LOC_Os09g29660 and LOC_Os09g29670), suggesting the possibility of relatively recent local gene duplication events. This analysis suggests that the common ancestor of Poaceae species possessed over 30 ABCG-like genes and that further expansion of the gene family occurred during subsequent lineage diversification.

## 4. Conclusions

In this study, colocation between the *Lp*ABCG5 gene and the plant type QTL on perennial ryegrass LG4 was demonstrated, as predicted by the comparative approach. Further characterisation suggested that ABCG5 orthologues are functionally conserved as plant architecture-controlling genes in grass and cereal species. The *Lp*ABCG6 gene, which is the most similar paralogue to the *Lp*ABCG5 gene at the DNA sequence level, was also identified, and additional ABCG gene candidates were identified through deep-sequencing of tissue-specific cDNA libraries. The other ABCG genes may be related to the molecular function of the *Lp*ABCG5 gene through heterodimer interaction. Phylogenetic analysis has further clarified the processes of ABCG gene family diversification within the Poaceae family, revealing the presence of both taxonomically conserved and species-specific genes.

Comparative genomics approaches have identified a number of conserved genes as candidates for agronomically important traits, including *Lp*ABCG5 as a candidate for plant type variation, as shown in this study. Additional strategies, however, may be required for further understanding of such complex traits, as species-unique genes may be involved in the mechanisms. Although a reduced plant size has been a major objective for crop breeding for several decades [41, 42], the emerging demand for grass crop species, such as sorghum and maize, as renewable energy resources requires an alternative breeding strategy to deliver increases in plant size [43]. The known phenotype of putative loss-of-function mutations in *Os*ABCG5 suggests the possibility that manipulation of the orthologues in biomass species, such as by transgenic overexpression, may generate desirable characteristics [19, 21].

## Figures and Tables

**Table 1:** Identity and classification of rice gene sequences employed for comparative genomic analysis.

TIGR UI*	RAP-DB UI**	Other name		Reference
LOC_Os03g17350	Os03g0281900	*Os*ABCG5	ABC transporter-related domain containing protein	[19, 20]
LOC_Os03g17370	Os03g0282100	*Os*ABCG6	Putative ABC transporter family protein	[20]
LOC_Os03g17300	Os03g0281500		Resistance protein candidate	
LOC_Os03g17310	Os03g0281600		Ca^2+^-ATPase	
LOC_Os03g17340	Os03g0281800		Sulfated surface glycoprotein 185 precursor	
LOC_Os03g17410	Os03g0282300		Conserved hypothetical protein	

*Unique identifier in TIGR database.

**Unique identifier in the rice annotation project database (RAP-DB).

**Table 2:** Putative perennial ryegrass ABCG genes identified through transcriptome sequencing. Highest-matching genes in rice are shown as candidate orthologues, with *E* values indicating level of similarity with the perennial ryegrass sequences.

Sequence name	Tissue	Candidate rice orthologue		*E* value
		TIGR UI	RAP-DB UI	
LpABCG1_cand	Leaf	LOC_01g03144	Os01g0121600	3.00*E*−72
LpABCG9_cand	Leaf	LOC_04g44610	Os04g0528300	4.00*E*−37
LpABCG12_cand	Leaf	LOC_05g13520	Os05g0222200	2.00*E*−29
LpABCG16_cand	Leaf, Anther	LOC_06g51460	Os06g0731200	3.00*E*−129
LpABCG18_cand	Leaf	LOC_08g07010	Os08g0167000	5.00*E*−36
LpABCG25_cand	Pistil	LOC_10g30610	Os10g0442900	5.00*E*−66
LpABCG26_cand	Anther	LOC_10g35180	Os10g0494300	8.00*E*−104
LpABCG27_cand	Leaf	LOC_11g07600	Os11g0177400	6.00*E*−54
LpABCG28_cand	Anther	LOC_11g22350	Os11g0416900	8.00*E*−68
LpABCG30_cand	Leaf	LOC_12g22284	Os12g0411700	2.00*E*−78
LpABCG31_cand	Anther	LOC_01g08260	Os01g0177900	6.00*E*−24
LpABCG41_cand	Leaf	LOC_02g32690	Os02g0528900	5.00*E*−48
LpABCG45_cand	Anther	LOC_08g43120	Os08g0544400	1.00*E*−42
LpABCG47_cand	Pistil	LOC_09g16380	Os09g0333000	4.00*E*−40
